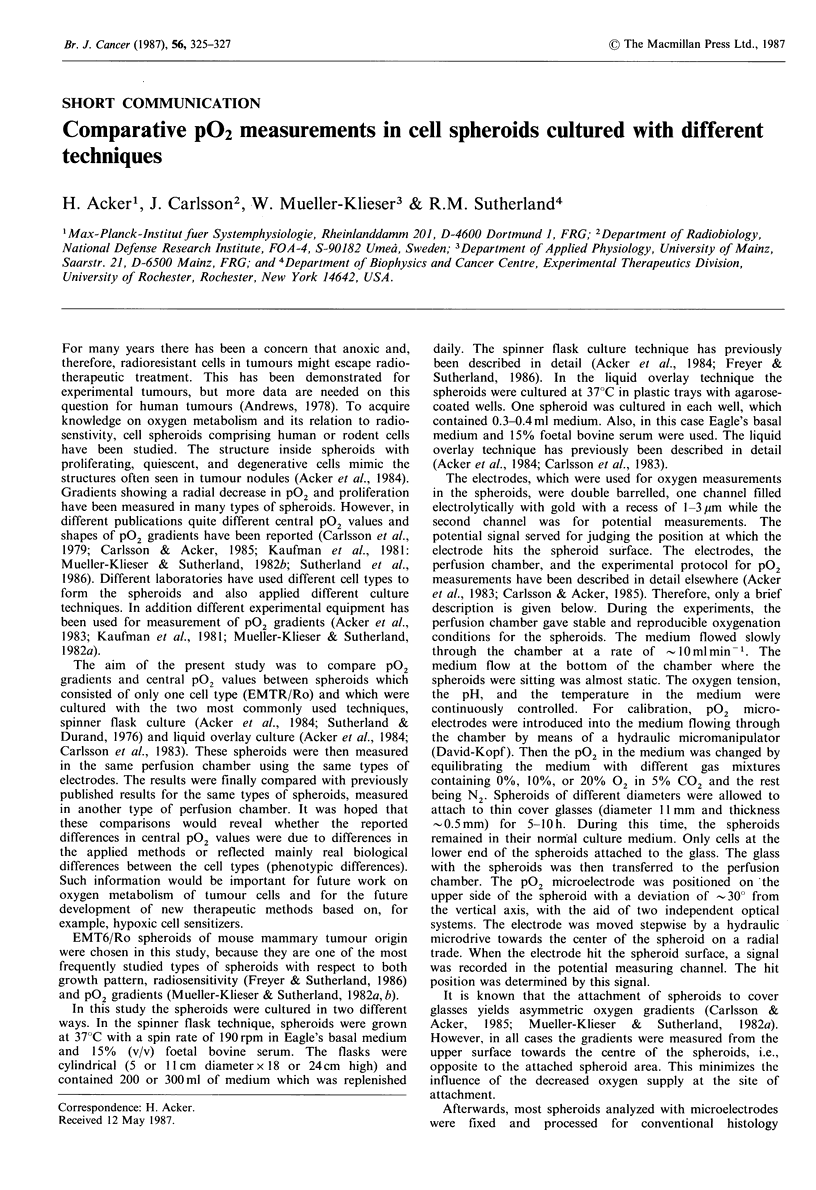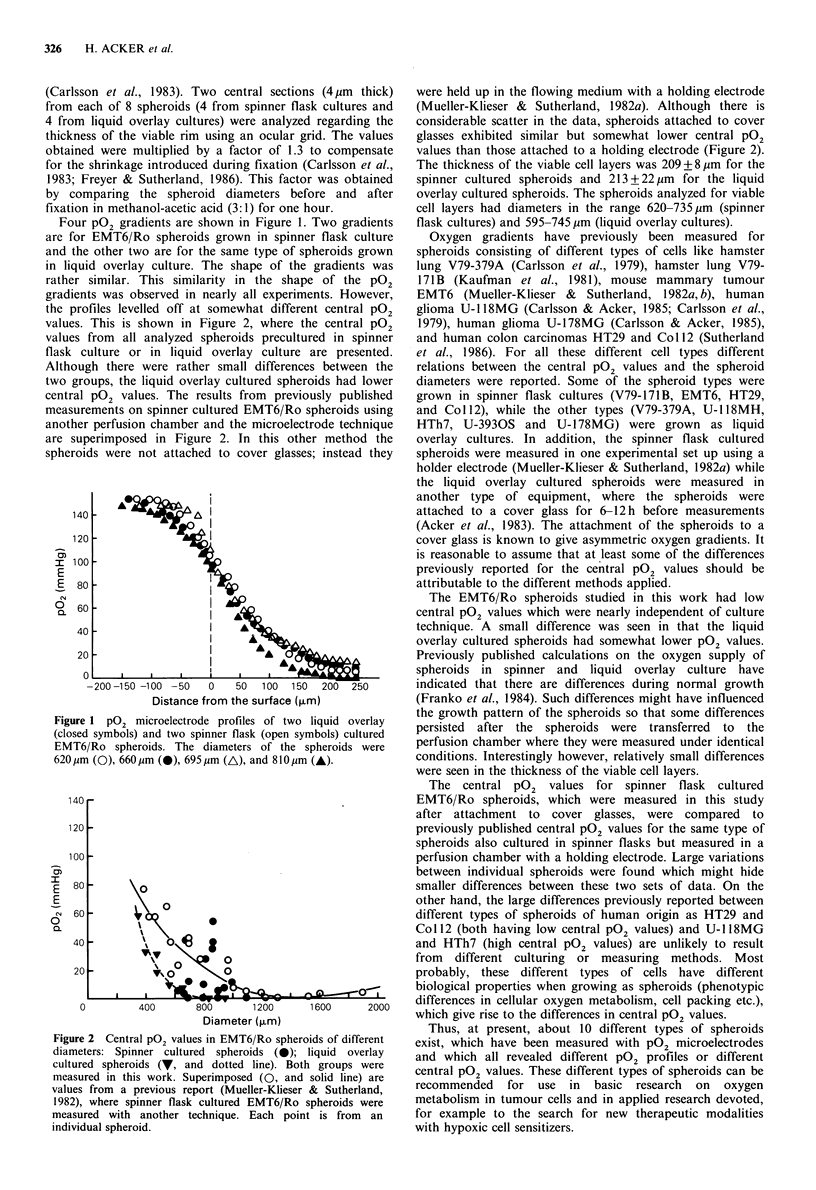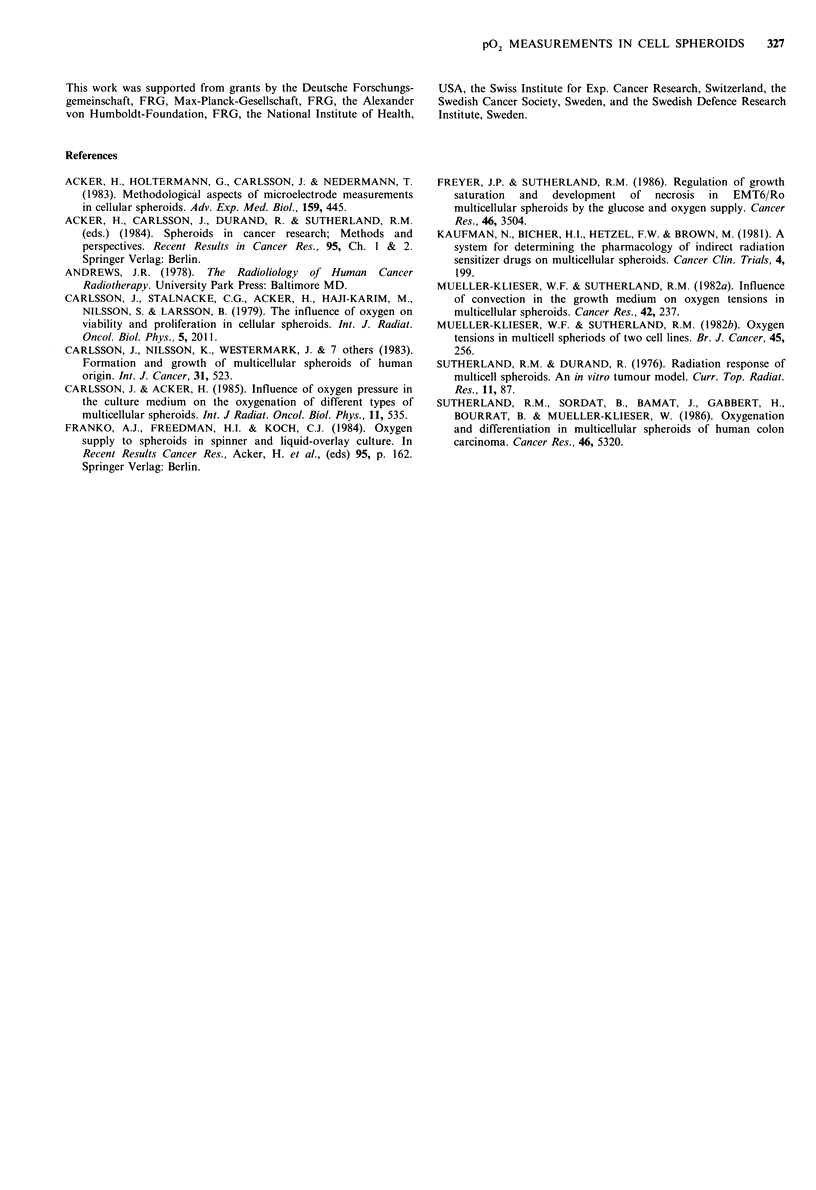# Comparative pO2 measurements in cell spheroids cultured with different techniques.

**DOI:** 10.1038/bjc.1987.197

**Published:** 1987-09

**Authors:** H. Acker, J. Carlsson, W. Mueller-Klieser, R. M. Sutherland

**Affiliations:** Max-Planck-Institut fuer Systemphysiologie, Dortmund, FRG.


Br. J. Cancer (1987), 56, 325–327 © The Macmillan Press Ltd., 1987

SHORT COMMUNICATION

### Comparative $pO_2$ measurements in cell spheroids cultured with different techniques

H. Acker[1], J. Carlsson[2], W. Mueller-Klieser[3] & R.M. Sutherland[4]

[1]*Max-Planck-Institut fuer Systemphysiologie, Rheinlanddamm 201, D-4600 Dortmund 1, FRG;* [2]*Department of Radiobiology, National Defense Research Institute, FOA-4, S-90182 Umeå, Sweden;* [3]*Department of Applied Physiology, University of Mainz, Saarstr. 21, D-6500 Mainz, FRG; and* [4]*Department of Biophysics and Cancer Centre, Experimental Therapeutics Division, University of Rochester, Rochester, New York 14642, USA.*

For many years there has been a concern that anoxic and, therefore, radioresistant cells in tumours might escape radiotherapeutic treatment. This has been demonstrated for experimental tumours, but more data are needed on this question for human tumours (Andrews, 1978). To acquire knowledge on oxygen metabolism and its relation to radiosenstivity, cell spheroids comprising human or rodent cells have been studied. The structure inside spheroids with proliferating, quiescent, and degenerative cells mimic the structures often seen in tumour nodules (Acker *et al.*, 1984). Gradients showing a radial decrease in $pO_2$ and proliferation have been measured in many types of spheroids. However, in different publications quite different central $pO_2$ values and shapes of $pO_2$ gradients have been reported (Carlsson *et al.*, 1979; Carlsson & Acker, 1985; Kaufman *et al.*, 1981: Mueller-Klieser & Sutherland, 1982*b*; Sutherland *et al.*, 1986). Different laboratories have used different cell types to form the spheroids and also applied different culture techniques. In addition different experimental equipment has been used for measurement of $pO_2$ gradients (Acker *et al.*, 1983; Kaufman *et al.*, 1981; Mueller-Klieser & Sutherland, 1982*a*).

The aim of the present study was to compare $pO_2$ gradients and central $pO_2$ values between spheroids which consisted of only one cell type (EMTR/Ro) and which were cultured with the two most commonly used techniques, spinner flask culture (Acker *et al.*, 1984; Sutherland & Durand, 1976) and liquid overlay culture (Acker *et al.*, 1984; Carlsson *et al.*, 1983). These spheroids were then measured in the same perfusion chamber using the same types of electrodes. The results were finally compared with previously published results for the same types of spheroids, measured in another type of perfusion chamber. It was hoped that these comparisons would reveal whether the reported differences in central $pO_2$ values were due to differences in the applied methods or reflected mainly real biological differences between the cell types (phenotypic differences). Such information would be important for future work on oxygen metabolism of tumour cells and for the future development of new therapeutic methods based on, for example, hypoxic cell sensitizers.

EMT6/Ro spheroids of mouse mammary tumour origin were chosen in this study, because they are one of the most frequently studied types of spheroids with respect to both growth pattern, radiosensitivity (Freyer & Sutherland, 1986) and $pO_2$ gradients (Mueller-Klieser & Sutherland, 1982*a*, *b*).

In this study the spheroids were cultured in two different ways. In the spinner flask technique, spheroids were grown at 37°C with a spin rate of 190 rpm in Eagle's basal medium and 15% (v/v) foetal bovine serum. The flasks were cylindrical (5 or 11 cm diameter × 18 or 24 cm high) and contained 200 or 300 ml of medium which was replenished daily. The spinner flask culture technique has previously been described in detail (Acker *et al.*, 1984; Freyer & Sutherland, 1986). In the liquid overlay technique the spheroids were cultured at 37°C in plastic trays with agarose-coated wells. One spheroid was cultured in each well, which contained 0.3–0.4 ml medium. Also, in this case Eagle's basal medium and 15% foetal bovine serum were used. The liquid overlay technique has previously been described in detail (Acker *et al.*, 1984; Carlsson *et al.*, 1983).

The electrodes, which were used for oxygen measurements in the spheroids, were double barrelled, one channel filled electrolytically with gold with a recess of 1–3 $\mu$m while the second channel was for potential measurements. The potential signal served for judging the position at which the electrode hits the spheroid surface. The electrodes, the perfusion chamber, and the experimental protocol for $pO_2$ measurements have been described in detail elsewhere (Acker *et al.*, 1983; Carlsson & Acker, 1985). Therefore, only a brief description is given below. During the experiments, the perfusion chamber gave stable and reproducible oxygenation conditions for the spheroids. The medium flowed slowly through the chamber at a rate of $\sim 10\,ml\,min^{-1}$. The medium flow at the bottom of the chamber where the spheroids were sitting was almost static. The oxygen tension, the pH, and the temperature in the medium were continuously controlled. For calibration, $pO_2$ microelectrodes were introduced into the medium flowing through the chamber by means of a hydraulic micromanipulator (David-Kopf). Then the $pO_2$ in the medium was changed by equilibrating the medium with different gas mixtures containing 0%, 10%, or 20% $O_2$ in 5% $CO_2$ and the rest being $N_2$. Spheroids of different diameters were allowed to attach to thin cover glasses (diameter 11 mm and thickness ~0.5 mm) for 5–10 h. During this time, the spheroids remained in their normal culture medium. Only cells at the lower end of the spheroids attached to the glass. The glass with the spheroids was then transferred to the perfusion chamber. The $pO_2$ microelectrode was positioned on the upper side of the spheroid with a deviation of ~30° from the vertical axis, with the aid of two independent optical systems. The electrode was moved stepwise by a hydraulic microdrive towards the center of the spheroid on a radial trade. When the electrode hit the spheroid surface, a signal was recorded in the potential measuring channel. The hit position was determined by this signal.

It is known that the attachment of spheroids to cover glasses yields asymmetric oxygen gradients (Carlsson & Acker, 1985; Mueller-Klieser & Sutherland, 1982*a*). However, in all cases the gradients were measured from the upper surface towards the centre of the spheroids, i.e., opposite to the attached spheroid area. This minimizes the influence of the decreased oxygen supply at the site of attachment.

Afterwards, most spheroids analyzed with microelectrodes were fixed and processed for conventional histology

Correspondence: H. Acker.
Received 12 May 1987.

(Carlsson *et al.*, 1983). Two central sections (4 μm thick) from each of 8 spheroids (4 from spinner flask cultures and 4 from liquid overlay cultures) were analyzed regarding the thickness of the viable rim using an ocular grid. The values obtained were multiplied by a factor of 1.3 to compensate for the shrinkage introduced during fixation (Carlsson *et al.*, 1983; Freyer & Sutherland, 1986). This factor was obtained by comparing the spheroid diameters before and after fixation in methanol-acetic acid (3:1) for one hour.

Four $pO_2$ gradients are shown in Figure 1. Two gradients are for EMT6/Ro spheroids grown in spinner flask culture and the other two are for the same type of spheroids grown in liquid overlay culture. The shape of the gradients was rather similar. This similarity in the shape of the $pO_2$ gradients was observed in nearly all experiments. However, the profiles levelled off at somewhat different central $pO_2$ values. This is shown in Figure 2, where the central $pO_2$ values from all analyzed spheroids precultured in spinner flask culture or in liquid overlay culture are presented. Although there were rather small differences between the two groups, the liquid overlay cultured spheroids had lower central $pO_2$ values. The results from previously published measurements on spinner cultured EMT6/Ro spheroids using another perfusion chamber and the microelectrode technique are superimposed in Figure 2. In this other method the spheroids were not attached to cover glasses; instead they were held up in the flowing medium with a holding electrode (Mueller-Klieser & Sutherland, 1982*a*). Although there is considerable scatter in the data, spheroids attached to cover glasses exhibited similar but somewhat lower central $pO_2$ values than those attached to a holding electrode (Figure 2). The thickness of the viable cell layers was $209 \pm 8$ μm for the spinner cultured spheroids and $213 \pm 22$ μm for the liquid overlay cultured spheroids. The spheroids analyzed for viable cell layers had diameters in the range 620–735 μm (spinner flask cultures) and 595–745 μm (liquid overlay cultures).

**Figure 1** $pO_2$ microelectrode profiles of two liquid overlay (closed symbols) and two spinner flask (open symbols) cultured EMT6/Ro spheroids. The diameters of the spheroids were 620 μm (○), 660 μm (●), 695 μm (△), and 810 μm (▲).

**Figure 2** Central $pO_2$ values in EMT6/Ro spheroids of different diameters: Spinner cultured spheroids (●); liquid overlay cultured spheroids (▼, and dotted line). Both groups were measured in this work. Superimposed (○, and solid line) are values from a previous report (Mueller-Klieser & Sutherland, 1982), where spinner flask cultured EMT6/Ro spheroids were measured with another technique. Each point is from an individual spheroid.

Oxygen gradients have previously been measured for spheroids consisting of different types of cells like hamster lung V79-379A (Carlsson *et al.*, 1979), hamster lung V79-171B (Kaufman *et al.*, 1981), mouse mammary tumour EMT6 (Mueller-Klieser & Sutherland, 1982*a*,*b*), human glioma U-118MG (Carlsson & Acker, 1985; Carlsson *et al.*, 1979), human glioma U-178MG (Carlsson & Acker, 1985), and human colon carcinomas HT29 and Co112 (Sutherland *et al.*, 1986). For all these different cell types different relations between the central $pO_2$ values and the spheroid diameters were reported. Some of the spheroid types were grown in spinner flask cultures (V79-171B, EMT6, HT29, and Co112), while the other types (V79-379A, U-118MH, HTh7, U-393OS and U-178MG) were grown as liquid overlay cultures. In addition, the spinner flask cultured spheroids were measured in one experimental set up using a holder electrode (Mueller-Klieser & Sutherland, 1982*a*) while the liquid overlay cultured spheroids were measured in another type of equipment, where the spheroids were attached to a cover glass for 6–12 h before measurements (Acker *et al.*, 1983). The attachment of the spheroids to a cover glass is known to give asymmetric oxygen gradients. It is reasonable to assume that at least some of the differences previously reported for the central $pO_2$ values should be attributable to the different methods applied.

The EMT6/Ro spheroids studied in this work had low central $pO_2$ values which were nearly independent of culture technique. A small difference was seen in that the liquid overlay cultured spheroids had somewhat lower $pO_2$ values. Previously published calculations on the oxygen supply of spheroids in spinner and liquid overlay culture have indicated that there are differences during normal growth (Franko *et al.*, 1984). Such differences might have influenced the growth pattern of the spheroids so that some differences persisted after the spheroids were transferred to the perfusion chamber where they were measured under identical conditions. Interestingly however, relatively small differences were seen in the thickness of the viable cell layers.

The central $pO_2$ values for spinner flask cultured EMT6/Ro spheroids, which were measured in this study after attachment to cover glasses, were compared to previously published central $pO_2$ values for the same type of spheroids also cultured in spinner flasks but measured in a perfusion chamber with a holding electrode. Large variations between individual spheroids were found which might hide smaller differences between these two sets of data. On the other hand, the large differences previously reported between different types of spheroids of human origin as HT29 and Co112 (both having low central $pO_2$ values) and U-118MG and HTh7 (high central $pO_2$ values) are unlikely to result from different culturing or measuring methods. Most probably, these different types of cells have different biological properties when growing as spheroids (phenotypic differences in cellular oxygen metabolism, cell packing etc.), which give rise to the differences in central $pO_2$ values.

Thus, at present, about 10 different types of spheroids exist, which have been measured with $pO_2$ microelectrodes and which all revealed different $pO_2$ profiles or different central $pO_2$ values. These different types of spheroids can be recommended for use in basic research on oxygen metabolism in tumour cells and in applied research devoted, for example to the search for new therapeutic modalities with hypoxic cell sensitizers.

This work was supported from grants by the Deutsche Forschungsgemeinschaft, FRG, Max-Planck-Gesellschaft, FRG, the Alexander von Humboldt-Foundation, FRG, the National Institute of Health, USA, the Swiss Institute for Exp. Cancer Research, Switzerland, the Swedish Cancer Society, Sweden, and the Swedish Defence Research Institute, Sweden.

**References**

ACKER, H., HOLTERMANN, G., CARLSSON, J. & NEDERMANN, T. (1983). Methodological aspects of microelectrode measurements in cellular spheroids. *Adv. Exp. Med. Biol.*, **159**, 445.

ACKER, H., CARLSSON, J., DURAND, R. & SUTHERLAND, R.M. (eds.) (1984). Spheroids in cancer research; Methods and perspectives. *Recent Results in Cancer Res.*, **95**, Ch. 1 & 2. Springer Verlag: Berlin.

ANDREWS, J.R. (1978). *The Radioliology of Human Cancer Radiotherapy*. University Park Press: Baltimore MD.

CARLSSON, J., STALNACKE, C.G., ACKER, H., HAJI-KARIM, M., NILSSON, S. & LARSSON, B. (1979). The influence of oxygen on viability and proliferation in cellular spheroids. *Int. J. Radiat. Oncol. Biol. Phys.*, **5**, 2011.

CARLSSON, J., NILSSON, K., WESTERMARK, J. & 7 others (1983). Formation and growth of multicellular spheroids of human origin. *Int. J. Cancer*, **31**, 523.

CARLSSON, J. & ACKER, H. (1985). Influence of oxygen pressure in the culture medium on the oxygenation of different types of multicellular spheroids. *Int. J Radiat. Oncol. Biol. Phys.*, **11**, 535.

FRANKO, A.J., FREEDMAN, H.I. & KOCH, C.J. (1984). Oxygen supply to spheroids in spinner and liquid-overlay culture. In *Recent Results Cancer Res.*, Acker, H. *et al.*, (eds) **95**, p. 162. Springer Verlag: Berlin.

FREYER, J.P. & SUTHERLAND, R.M. (1986). Regulation of growth saturation and development of necrosis in EMT6/Ro multicellular spheroids by the glucose and oxygen supply. *Cancer Res.*, **46**, 3504.

KAUFMAN, N., BICHER, H.I., HETZEL, F.W. & BROWN, M. (1981). A system for determining the pharmacology of indirect radiation sensitizer drugs on multicellular spheroids. *Cancer Clin. Trials*, **4**, 199.

MUELLER-KLIESER, W.F. & SUTHERLAND, R.M. (1982*a*). Influence of convection in the growth medium on oxygen tensions in multicellular spheroids. *Cancer Res.*, **42**, 237.

MUELLER-KLIESER, W.F. & SUTHERLAND, R.M. (1982*b*). Oxygen tensions in multicell spheriods of two cell lines. *Br. J. Cancer*, **45**, 256.

SUTHERLAND, R.M. & DURAND, R. (1976). Radiation response of multicell spheroids. An *in vitro* tumour model. *Curr. Top. Radiat. Res.*, **11**, 87.

SUTHERLAND, R.M., SORDAT, B., BAMAT, J., GABBERT, H., BOURRAT, B. & MUELLER-KLIESER, W. (1986). Oxygenation and differentiation in multicellular spheroids of human colon carcinoma. *Cancer Res.*, **46**, 5320.